# Tertiary lymphoid structures in neuroinflammation coordinate neuroimmune homeostasis and pathological progression

**DOI:** 10.1186/s12974-026-03961-6

**Published:** 2026-07-10

**Authors:** Yan Zhang, Peishen Han, Xiaoling Zhang

**Affiliations:** 1 Key Laboratory of Organ Regeneration and Transplantation of Ministry of Education, First Hospital of Jilin University, Changchun, China; 2 National-Local Joint Engineering Laboratory of Animal Models for Human Disease, First Hospital of Jilin University, Changchun, China

**Keywords:** Tertiary lymphoid structures, Neuroinflammation, Neuroimmune interaction, Neuroimmune homeostasis, Disease progression

## Abstract

The central nervous system (CNS) has long been considered immune privilege due to the blood-brain barrier, lack of traditional lymphatic drainage, and unique immune microenvironment. However, recent neuroimmunology research has demonstrated that the CNS maintains continuous communication with the peripheral immune system via meningeal lymphatic vessels, lymphoid systems, and border-associated macrophages. This paradigm shift has brought tertiary lymphoid structures (TLSs), ectopic lymphoid aggregates induced by chronic inflammation, infection, or tumors, into focus as key players in neuroimmune interactions. TLSs exert a dual effect in neuroinflammation. In infectious diseases like viral encephalitis, they promote local antibody production and T cell responses, aiding pathogen clearance. In contrast, in multiple sclerosis, autoimmune encephalitis, Alzheimer’s disease, and Parkinson’s disease, TLSs may sustain chronic inflammation, drive autoantibody production, and accelerate neurodegeneration. This review systematically summarizes the composition, induction mechanisms, and functional heterogeneity of TLSs across neurological diseases. We discuss their protective versus pathogenic roles in neuroinflammation and highlight their diagnostic value and therapeutic potential, aiming to provide new insights for precision intervention in neuroimmunological disorders.

## Introduction

### The evolution of central nervous system immunity from exemption to precision surveillance

**Fig. 1:**
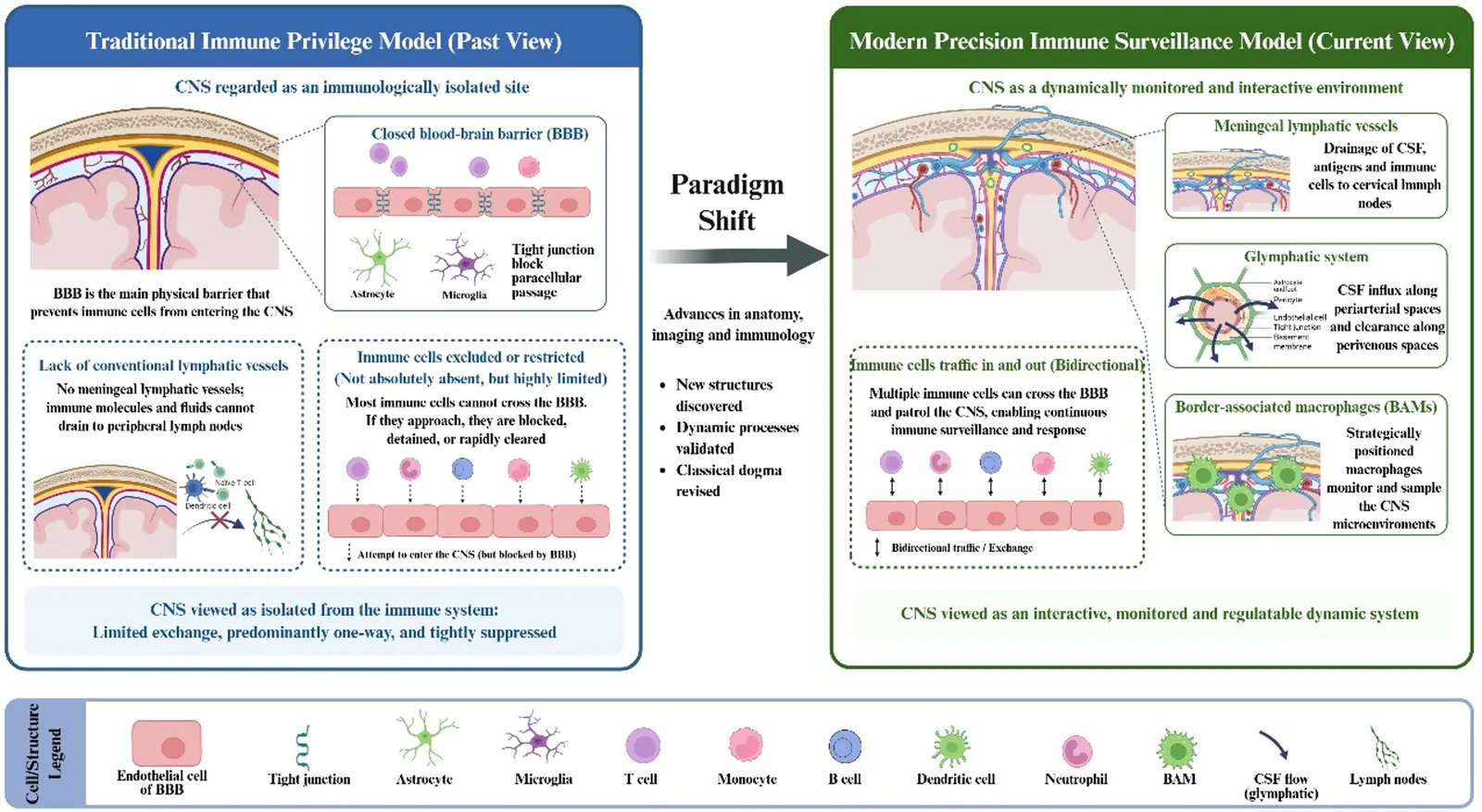
Evolution of the central nervous system immune concept from immune privilege to precision immune surveillance. The left panel illustrates the classical immune privilege model of the central nervous system (CNS), in which the blood-brain barrier (BBB), absence of conventional lymphatic vessels, and restricted immune cell entry contributed to the concept of the CNS as an immunologically isolated site. The right panel depicts the modern precision immune surveillance model, incorporating recently identified structures and processes including meningeal lymphatic vessels, the glymphatic system, and border-associated macrophages (BAMs). These components collectively facilitate bidirectional trafficking of CSF, antigens, and immune cells, enabling continuous immune monitoring and regulation within the CNS. The central arrow indicates the paradigm shift from immune privilege toward dynamic immune surveillance and precision regulation. This schematic diagram is a conceptual framework proposed based on existing evidence

Traditional thinking has long regarded the central nervous system (CNS) as an immune privilege region, mainly due to the presence of the blood-brain barrier (BBB), the lack of traditional lymphatic drainage, and its immunosuppressive environment. It is believed that nerve tissue is relatively isolated from the peripheral immune system, which shields it from inflammatory damage [1]. However, this view has been gradually revised, and new research shows that the CNS is not completely isolated, but rather communicates and monitors the peripheral immune system through sophisticated mechanisms [2, 3]. The discovery of lymphatic vessels in the meninges is one of the most surprising findings in neuroimmunology in recent years. This discovery overturns the idea that the brain is immune-isolated, demonstrating a direct pathway for the migration of immune cells and fluid exchange between the brain and the peripheral immune system^2^. Meanwhile, the close connection between the meningeal lymphatic vessels and the lymphoid system enables the clearance of metabolic waste, the transport of immune cells, and CNS immune surveillance [4]. Dysfunction of the meningeal lymphatic system can lead to the accumulation of toxic proteins in the brain and contribute to the development of a range of neurodegenerative diseases, such as Alzheimer’s disease (AD) and Parkinson’s disease (PD) [5, 6]. In the brain parenchyma, microglia are considered to be the main resident cells, while there are several other specialized macrophage populations at the boundary of the central nervous system, including dura mater, pia mater, perivascular and choroid plexus macrophages, collectively referred to as central nervous system-associated macrophages (CAMs) or boundary-associated macrophages (BAMs). They are an important part of the communication between the CNS and the peripheral immune system. They can present antigens, regulate T cell infiltration, and maintain the immune balance of the CNS in homeostasis and disease states [7]. The immune paradigm of the central nervous system has evolved from the traditional concept of immune exemption into a highly complex and sophisticated immune surveillance system, in which meningeal lymphatic vessels (MLVs), glymphatic systems, and BAMs are key components [3, 8, 9] (Fig. 1).

It is worth noting that the function of MLVs is not limited to waste clearance and antigen drainage. The structural remodeling and functional status of lymphatic vessels can directly regulate the local immune microenvironment, thereby affecting the aggregation and organization of immune cells, and this process is a key link in the formation of TLS. Therefore, TLS does not emerge in isolation, but rather forms within a broader lymphatic and stromal ecosystem. In melanoma studies, lymphatic vessels shape the local inflammatory microenvironment by regulating cytokine expression and immune cell infiltration [10]. When lymphatic function is impaired, an immunosuppressive microenvironment can be formed and disease progression can be accelerated [11, 12]. These findings suggest that MLVs may regulate local immune responses through similar mechanisms in the CNS, providing an important conceptual basis for understanding the formation of TLS in the CNS. Future research needs to further elucidate how MLVs regulate the initiation, maturation, and functional orientation of TLS in CNS through their drainage function and molecular signaling.

### Neuroinflammation from physiological homeostasis to chronic pathogenic inflammation

This paradigm shift has profoundly changed our understanding of neuroinflammation, from initially considering it as part of physiological homeostasis to now recognizing it as a potential chronic pathogenic inflammation in disease. Neuroinflammation is a complex and dynamic response of the central nervous system to injury, infection or disease [13] involving interactions between various cellular and molecular components within it, primarily mediated by immune cells inherent to the central nervous system, such as microglia and astrocytes, as well as immune cells infiltrated by the peripheral immune system [14], and the pro-inflammatory cytokines, chemokines and other factors produced by them also play a central role in chronic neuroinflammation. Neuroinflammation has traditionally been regarded as a defense mechanism, but it is now recognized as having a dual nature: while it has neuroprotective effects, promotes pathogen clearance and tissue repair to maintain central nervous system homeostasis, its chronic or dysregulated form may turn into neurotoxic effects, significantly promoting the progression of neurodegenerative diseases such as Alzheimer’s disease, Parkinson’s disease and multiple sclerosis (MS) and exacerbating neurological dysfunction [15, 16].

### Key cells and classic inducing factors in the tertiary lymphoid structure

Tertiary lymphoid structures (TLSs) are ectopic lymphoid aggregates that form in non-lymphoid tissues under chronic inflammatory conditions. They are also referred to as ectopic lymphoid tissues, although this term broadly encompasses a spectrum of lymphoid aggregates ranging from simple lymphocyte clusters to highly organized structures. In this review, we use “TLS” to refer specifically to the more organized end of this spectrum, whereas less organized aggregates are described as “TLS-like structures” or “lymphoid aggregates”. Fully developed TLS have structures and functions similar to secondary lymphoid organs (SLOs), especially in terms of lymph nodes [17]. TLSs have a similar structure to SLOs but lack a capsule and consist of separate B cell and T cell regions containing follicular dendritic cells (FDCs), and high endothelial venules (HEVs). Germinal centers (GCs) are found only in a subset of mature TLS, reflecting the highest degree of TLS organization [18–20]. B cells in TLSs can form follicle-like structures, which then mature into germinal centers, where affinity maturation and class switching occur, producing high-affinity antibodies against specific antigens [19, 21]. T cells, especially effector T cells and follicular helper T cells (Tfh), form independent T cell regions in TLSs and play a key role in adaptive immune responses. The T cell regions also provide helper signals to CD8^+^ T cells, promoting cytotoxic immune responses [22, 23]. FDCs are the structural and functional building blocks of B cell follicles in TLSs. They capture and present antigen-antibody complexes through Fc receptors and complement receptors on their surface, and secrete the chemokine CXCL13 to attract B cell aggregation. They are an important indicator of TLS maturity [23, 24]. HEVs are an important marker of lymphocytes in TLSs. These special vascular structures can efficiently mediate the entry of lymphocytes from the blood into non-lymphoid tissues. Their endothelial cells express peripheral lymph node addressin (PNAd), which binds to L-selectin on the surface of lymphocytes, promoting lymphocyte recruitment and playing a crucial role in the function of TLSs [21, 24].

**Fig. 2:**
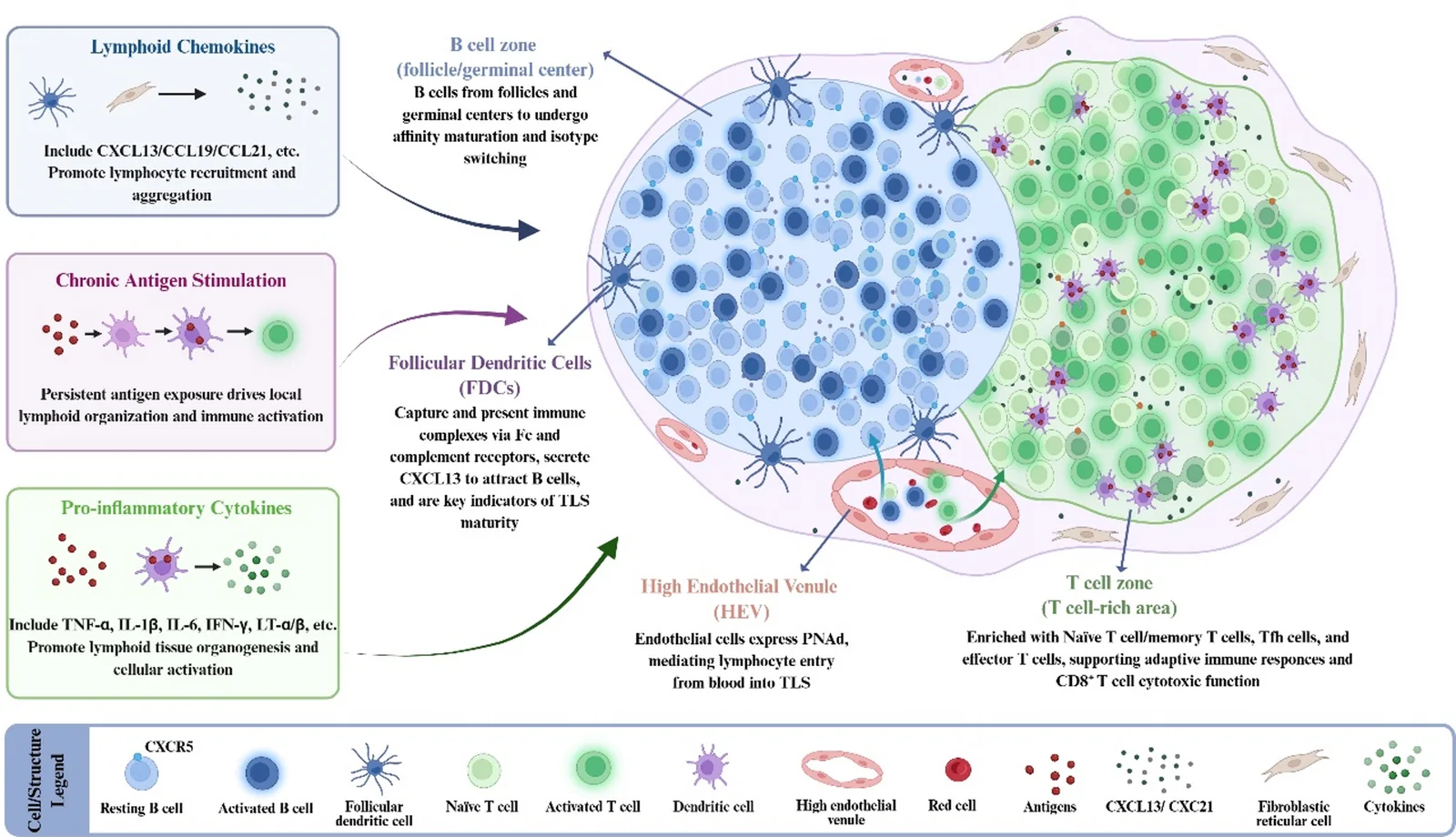
Composition and induction of tertiary lymphoid structures. The core section (right panel) illustrates the typical microarchitecture of TLSs, including B-cell follicles/germinal centers, T-cell zones, FDCs, and high endothelial venules (HEVs). B-cell regions support B-cell activation, affinity maturation, and class-switch recombination, whereas T-cell zones are enriched with helper and effector T cells that coordinate adaptive immune responses. FDCs maintain follicular organization through immune complex presentation and CXCL13 secretion, while HEVs mediate lymphocyte recruitment from the circulation into TLSs. Surrounding arrows indicate the major factors driving TLS formation, including lymphoid chemokines (CXCL13, CCL19, and CCL21), persistent antigen stimulation, and pro-inflammatory cytokines such as TNF-α, IL-1β, IL-6, IFN-γ, and LTα/β. Together, these signals promote ectopic lymphoid neogenesis and local immune activation. This diagram is an integrated model for TLS formation based on existing evidence. Further clarification is needed on how these factors are sequentially activated and perform their respective roles in different structural regions during TLS formation

The formation of TLSs is a dynamic lymphoid regeneration process that depends on the continuous interaction between hematopoietic cells and non-hematopoietic stromal cells, and its molecular mechanism is similar to that of the development of lymphoid organs during the embryonic period [23, 24]. Classic factors that induce the formation of TLSs include lymphokines and chronic antigen stimulation. Chemokines play a central role in the formation and organization of TLSs: CXCL13 is mainly secreted by FDCs and attracts B cells to form B cell follicles through the chemokine receptor CXCR5 [25]; CCL19/CCL21 is mainly secreted by fibroblast reticulocytes (FRCs) and attracts T cells and dendritic cells (DCs) into the T cell region through the chemokine receptor CCR7, thereby ensuring that B cells and T cells form well-defined functional regions in TLSs [17, 23, 26]. The lymphotoxin (LT) signaling pathway, particularly the LTα1β2-LTβR axis, is a core signaling pathway for the initiation and maintenance of TLSs [22]. The membrane-bound form of lymphotoxin (LTα1β2) on the surface of lymphoid tissue inducer-like cells (LTi-like cells) binds to the lymphotoxin β receptor (LTβR) on the surface of stromal tissue cells, activating the non-canonical NF-κB signaling pathway, leading to stromal cell reprogramming, secretion of chemokines, and formation of the cellular scaffold required for lymphocyte homing. Chronic antigen stimulation is a key driver of TLS formation. Under pathological conditions such as chronic inflammation, infection, or tumors, TLS can trigger immune responses independent of SLOs [19]. Locally persistent antigens can activate dendritic cells, leading to the production of pro-inflammatory cytokines such as type I interferon, IL-6 and TNF-α. These factors can upregulate the expression of chemokines in stromal cells, further promoting the recruitment of lymphocytes and the formation of TLSs [22, 23] (Fig. 2).

### Graded evidence of TLS in neurological diseases

Specifically, based on the histological criteria for TLS defined earlier in this section, the evidence for TLS and related lymphoid structures in the neurological diseases discussed in this review can be classified into three levels. MS represents the condition with confirmed bona fide TLS, as meningeal lymphoid aggregates have been demonstrated to possess segregated B/T cell zones, FDCs, high endothelial venules, and germinal center-like activity. In Alzheimer’s disease, Parkinson’s disease, amyotrophic lateral sclerosis (ALS), and chronic inflammatory demyelinating polyneuropathy (CIDP), current evidence is limited to TLS-like aggregates or simple lymphoid infiltrates, and bona fide TLS have not been consistently demonstrated. In viral encephalitis and autoimmune encephalitis, the presence of TLS remains speculative, as direct histopathological evidence in human CNS tissue is currently lacking. This graded distinction informs the discussion of controversies and open questions that follows.

This review aims to systematically explore the formation mechanisms, functions, and opposing roles of the tertiary lymphoid structures in neurological diseases under different disease contexts. Traditional views hold that the nervous system is strictly regulated by the immune system to maintain homeostasis and prevent nerve damage; however, recent studies have shown that TLSs have been widely found in a variety of neurological diseases and play a complex and often dual role [27]. For example, in autoimmune diseases such as MS, TLSs may exacerbate inflammation and pathological processes [17]; while in tumor microenvironments such as gliomas, they may promote anti-tumor immunity and are associated with better prognosis [28]. This striking duality in their roles underscores the urgency and importance of gaining a deeper understanding of the formation mechanisms and functional heterogeneity of TLSs. By reviewing and analyzing these issues, this review aims to provide key insights into the complex landscape of neuroimmune interactions and lay a theoretical foundation for the future development of precision treatment strategies targeting TLSs based on the characteristics of the disease microenvironment.

## Formation and regulation of the tertiary lymphatic structure in the nervous system

Tertiary lymphoid structures are not physiological structures of the central nervous system, but rather immune tissues that form ectopically in the context of chronic inflammation, autoimmune diseases, neurodegenerative diseases, or certain infections and tumors. Their formation and regulation involve complex anatomical microenvironments, molecular triggers, and intercellular interactions [23, 26].

### Anatomical microenvironment of TLS formation

The anatomical distribution of TLS in the nervous system exhibits high regional specificity, showing significant differences between the CNS and the peripheral nervous system (PNS). This difference is due to fundamental differences in anatomical barriers, immune microenvironments, and disease mechanisms between the two systems. Therefore, the following discusses the distribution characteristics of TLS in CNS and PNS respectively. The CNS is protected by the blood-brain barrier and the blood-cerebrospinal fluid barrier, with immune responses strictly regulated, making parenchymal TLS extremely rare; while the PNS has a relatively weaker blood-nerve barrier, allowing immune cells to more easily enter, and TLS-like structures are more likely to form under inflammatory conditions. These differences suggest that treatment strategies for TLS in CNS and PNS may need to be considered differently.

#### Anatomical distribution of TLS in the CNS

TLS within the CNS parenchyma is extremely rare, and its occurrence is usually closely associated with severe chronic inflammatory states [29]. It is only occasionally found in terminal, long-term uncontrolled severe neuroinflammation, such as B lymphoid follicles in the brain parenchyma of progressive multiple sclerosis [30]. This means that the CNS parenchyma can usually effectively resist the formation of such ectopic immune structures, which may be related to a strict immune control mechanism [29].

The meninges (especially the pia mater and dura mater) and perivascular spaces are the most common and functionally active sites of CNS-related TLSs [26, 31]. In the meninges, blood vessels and lymphatic structures facilitate the transport of immune cells and communication between the central nervous system and peripheral tissues. The meninges also contain a variety of immune cell populations, including B cells, T cells, macrophages, type 2 innate lymphocytes, and so on. This immune-rich environment makes the meninges a key immune interface at the boundary of the central nervous system [32], and also constitutes the interface between the cerebrospinal fluid and the peripheral immune system, supporting the recruitment of B cells and T cells, antigen presentation, and the production of local antibodies [26, 33].

Following acute spinal cord injury, B cells and T cells accumulate locally within the spinal cord meninges, forming TLS-like structures, and continue to accumulate in the later stages of repair [31]. In MS patients, especially in those with progressive or non-remitting MS, leptomeningeal TLS has become a relatively common pathological feature. These ectopic lymphoid aggregates are often located in the leptomeninges, adjacent to so-called type Ⅲ subpial cortical lesions, and are associated with more severe gray matter damage and worse clinical prognosis [26].

#### Anatomical distribution of TLS in the peripheral nervous system (PNS)

Compared with CNS, there are relatively few studies on the anatomical location of TLS in the peripheral nervous system (PNS), which mainly focus on areas associated with inflammatory neuropathy [34]. The anatomical barrier of PNS, the blood-nerve barrier (BNB), has significantly different structural features [35]. The blood-nerve barrier is composed of endothelial cells and their tight junctions, basement membrane, and surrounding cells in the neurointima, but its permeability is higher than that of the blood-brain barrier, and it lacks the tight regulatory layer formed by the foot processes of astrocytes [36, 37]. This makes PNS more susceptible to the influence of immune cells and inflammatory factors in the circulatory system, making it easier to form lymphoid aggregation structures under inflammatory conditions. This anatomical difference may be an important reason why TLS like structures in PNS are relatively easier to observe in inflammatory neuropathy.

The dorsal root ganglion (DRG) is the location of the cell bodies of sensory neurons and is also a relatively weak area of the blood-nerve barrier in PNS. It is easily affected by inflammation and immune cell infiltration. In PNS inflammatory models such as experimental autoimmune neuritis, lymphocytes have been observed to accumulate in the DRG, suggesting that the DRG is a potential site for TLS formation [26]. TLS-like structures can also be found in major nerve trunks such as the sciatic nerve root, especially in patients with neuropathy associated with chronic inflammatory demyelinating polyneuropathy (CIDP) or Sjögren’s syndrome [38], where these ectopic lymphoid aggregates indicate persistent local immune activation. Intraneural inflammation is a characteristic feature of PNS disease, and these infiltrating foci may provide a microenvironment for the formation of TLS [39].

### Molecular triggers and cellular regulators

The formation and maintenance of TLS depend on a complex series of molecular signals and cellular interactions. Among them, CXCL13 is a major chemokine of B cells, responsible for driving the recruitment of B cells to TLSs regions. In neuroinflammation, CXCL13 is also thought to be able to recruit B cells into cerebrospinal fluid (CSF). CXCR5 is the corresponding receptor of CXCL13 and is expressed on B cells and follicular helper T cells, enabling these cells to migrate along the CXCL13 gradient and form follicular-like structures of B cells in TLSs [40]. CCL19 and CCL21 are chemokines produced by fibroblast reticulocytes (FRCs). They guide T cell and dendritic cells to aggregate in the T cell region via the CCR7 receptor and are crucial for the homing of T cells and DCs to the T cell region [17, 41, 42]. Furthermore, the lymphotoxin signaling pathway is a key initiation mechanism for the formation of TLSs [43]. Lymphoid tissue-induced cells (LTi cells), such as ILC3-like cells or activated T/B cells, express a membrane-bound form of the lymphotoxin heterotrimer LTα1β2 on their surface [44]. When LTα1β2 binds to the lymphotoxin β receptor (LTβR) on the surface of stromal tissue cells (such as perivascular fibroblast precursors), it activates downstream signaling pathways, inducing the differentiation of lymphoid tissue stromal cells and the generation of HEVs. This signal activates the non-classical NF-κB signaling pathway, generating a continuous signal that is crucial for driving cell differentiation that requires long-term stable gene expression [22, 45].

In recent years, studies have found that ILC2s are a key type of lymphoid tissue induced cell in TLS formation. Amisaki et al. have revealed the core role of IL-33 activated ILC2s in TLS neogenesis [46]. This study elucidates that the alarm cytokine IL-33 released by inflammatory tissues can activate KLRG1⁺ ILC2s expressing lymphotoxins (LT), which cooperate with LTβR⁺ myeloid tissue cells to drive TLS formation through the LT-LTβR signaling pathway. In a mouse model of pancreatic cancer, recombinant IL-33 can amplify intratumoral ILC2s and promote TLS maturation, from early lymphoid aggregates to mature TLS containing germinal centers. Interestingly, these ILC2s with lymphoid tissue regeneration ability can migrate from the intestine to the tumor site via the gut-vascular axis and are regulated by the gut microbiota, suggesting that systemic factors may be involved in the regulation of TLS formation. In human pancreatic cancer TLS, IL-33^+^ cells and ILC2s with lymphoid tissue regeneration potential were also detected, and are associated with better prognosis. This discovery reveals the IL-33-ILC2-TLS axis, a targeted pathway for lymphoid tissue regeneration, and suggests that the role of ILC2s in TLS formation may extend beyond the tumor domain, and is worth further exploration in neurological diseases.

ILC2s not only participate in the structural formation of TLS, but their functionality in TLS is also receiving increasing attention. Previous review has summarized the dual role of ILC2s in cancer immune surveillance [47]. On the one hand, IL-33 activated ILC2s can exert direct anti-tumor immune effects by recruiting dendritic cells and CD8⁺ T cell axis or recruiting eosinophils; On the other hand, in gastric cancer, breast cancer and other tumors, ILC2s may also create tumor promoting microenvironment by promoting type 2 immune response. This functional duality is highly dependent on signal integration in the local microenvironment, indicating that the functional orientation of ILC2s in TLS may dynamically change with disease type and TLS maturation stage.

Meanwhile, type I/II interferons enhance antigen presentation by upregulating MHC-I/II and co-stimulatory molecules and promote the expression of chemokines such as CXCL10. Continuous innate interferon is an important inducer of TLS formation [48].

### Antigen presentation and chronic stimulation

The TLS in the nervous system can locally initiate and maintain adaptive immune responses, which is closely related to continuous antigen stimulation and antigen presentation functions of various cells. Under conditions of neurological inflammation, microglia and astrocytes can upregulate major histocompatibility complex II molecules (MHC-II) and co-stimulatory molecules under stimulation such as IFN-γ or Toll-like receptor (TLR) activation, thereby gaining non-classical antigen presentation capabilities [26, 49]. This means they can capture and present antigens within the nervous system, activate T cells, and thus participate in the formation of TLS and the amplification of the immune response. Under chronic inflammatory and pathological conditions, peripheral dendritic cells infiltrate the nervous system and play a key antigen-presenting role within the TLS [50–52]. Infiltrating classical dendritic cells (cDC1/cDC2) provide classical MHC-I/MHC-II presentation and work synergistically with signals such as CD40L to initiate T cell and B cell responses. For example, studies have shown that type 1 dendritic cells (cDC1) can control the formation, maintenance and function of TLSs in cancer [53]. Continuous antigen stimulation is a necessary condition for the formation of TLSs [54]. In neurological diseases, multiple potential antigens can drive the clonal expansion of local lymphocytes and the formation of germinal centers. In autoimmune diseases such as MS, myelin-associated proteins (such as myelin oligodendrocyte glycoprotein and protein lipoprotein) can be presented as self-antigens and activate self-reactive lymphocytes, leading to the formation and maintenance of TLS [55, 56]. In addition, certain viral infections, such as EBV or HHV-6, may promote the occurrence of TLS in the nervous system through persistent viral antigen presentation [57, 58]. For example, the cytomegalovirus IE1/IE1mut therapeutic vaccine activates peripheral immunity and causes tumor regression by inducing TLS formation in glioblastoma [58]. In neurodegenerative diseases such as Alzheimer’s disease, the abnormal accumulation of misfolded proteins such as β-amyloid (Aβ) and α-synuclein may act as a persistent inflammatory stimulus and a potential antigen, driving chronic inflammation and TLS formation in the nervous system [59, 60].

### Matrix and vascular remodeling

The tissue structure of TLS requires precise matrix remodeling and angiogenesis, especially the formation of high endothelial venules, to support efficient recruitment and circulation of lymphocytes [61–63]. Under the regulation of LTβR and TGF-β signaling, PDGFRα^+^ fibroblasts can transdifferentiate into lymphoid stromal cells (LTo), which are the core components of TLS formation. They not only secrete extracellular matrix components to provide scaffolds for immune cells, but also support the structure and function of TLS by secreting chemokines and cytokines [62, 64]. Fibroblasts play an important role in infection, inflammation and cancer as immunomodulators, and their heterogeneity allows them to play different roles in maintaining immune homeostasis or driving immune responses [65]. NG2^+^ perivascular cells are widely distributed around blood vessels in the nervous system. Under the induction of LTβR and TGF-β signaling, they may also participate in the transdifferentiation into lymphoid stromal cells, thereby supporting the formation of TLS and the development of HEVs. The absence of Notch signaling in vascular endothelium has been shown to promote spontaneous formation of TLS in the kidney, liver and lung, suggesting the important regulatory role of perivascular cells and endothelial cells in TLS formation [61]. HEVs are crucial structures for the function of TLS, and they promote the efficient infiltration of lymphocytes from the blood into the TLS by expressing specific adhesion and chemokine molecules [63, 66]. The formation of HEVs is a highly regulated process that depends on the expression of VCAM-1/ICAM-1 adhesion molecules and PNAd on MECA-79^+^ high endothelial cells. This process is synergistically regulated by cytokines such as LTα/β, TNF, and IL-17. In sites of chronic inflammation, ordinary microvessels can transform into functional, HEV-like vessels, becoming a defining feature of TLS development [65].

The formation of TLS in the nervous system is a highly complex and dynamic process involving the synergistic effects of multiple cytokines and chemokines, as well as antigen presentation by resident glial cells and infiltrating immune cells. Meanwhile, stromal cells and vascular endothelial cells play a key role in remodeling the local microenvironment and forming HEVs, providing a structural basis for the recruitment of immune cells and the maintenance of TLS function.

## Protective role: the role of TLS in central nervous system immune surveillance and infection control

Before discussing the protective roles of TLS in specific infectious diseases, it is important to note that TLS functions are highly context-dependent. The same TLS structure that facilitates viral clearance in acute infection may, under conditions of persistent antigen stimulation or altered immune regulation, contribute to chronic inflammation or even autoimmunity. Thus, the “protective” designation described in this section refers to the predominant outcome reported in the literature under specific experimental or clinical conditions, and does not imply that TLS are universally or invariably beneficial in all infectious settings.

Although TLS is often discussed as a pathogenic factor in chronic inflammation and autoimmunity, increasing evidence suggests that TLS plays a key protective role in CNS immune surveillance and infection control [17, 67]. We will take the viral encephalitis model as an example to illustrate the protective mechanism of TLS (specific molecular and cellular mechanisms are described in Sect. "Multiple mechanisms by which TLS promotes viral clearance and long-term immune protection in the CNS").

### TLS in viral encephalitis

In viral encephalitis, it has been suggested that TLS within the CNS may form ectopically and play immune effector functions [68]. However, direct evidence for TLS in human patients is currently lacking, and their presence remains speculative. For example, in neurotropic virus infection models such as lymphocytic choriomeningitis virus (LCMV), persistent viral antigens can induce lymphocyte aggregation in the meninges and perivascular areas, forming TLS-like structures [69–71]. In this model, localized lymphoid tissue formation is associated with a more effective antiviral response, reflected in accelerated virus clearance [72, 73].

Viral encephalitis is a rare but serious syndrome caused by a variety of DNA and RNA viruses, such as herpes simplex virus, varicella-zoster virus, and RNA viruses from the Flaviviridae, Rhabdoviridae and Paramyxoviridae families [68, 69]. These viruses, once they invade the CNS, can cause serious neurological complications and even endanger life [74]. For a long time, the CNS has been considered an area with “immune exemption” and its immune response is strictly controlled to prevent nerve damage. However, studies have shown that immune-related structures and immune cell infiltration exist in the CNS and are involved in adaptive immunity [67]. In this context, the formation of TLSs is particularly important. As local immune command centers, they may achieve faster and more concentrated local immune defense by reducing reliance on peripheral lymphoid organs, and organize an efficient immune response when needed.

### Multiple mechanisms by which TLS promotes viral clearance and long-term immune protection in the CNS

#### Local clonal expansion and affinity maturation of antigen-specific B cells

Antigen-specific B cells entering the CNS can undergo efficient clonal expansion and affinity maturation at this location [71, 72]. In a viral encephalitis model, sequencing of B cell receptors in the CNS revealed local oligoclonal amplification, which strongly suggests the existence of an antigen-driven selection process. Unlike B cells in autoimmune diseases that mainly produce autoantibodies, in the context of viral infection, this process is mainly dedicated to producing high-affinity neutralizing antibodies against viral antigens, thereby effectively combating pathogens [70]. Follicular regulatory T cells (Tfr cells) regulate the germinal center B cell response in this process [71, 75, 76]. Some studies have shown that in mature TLS, B cells are selected and expanded, which produce antibodies [72].

#### Intrathecal antibody synthesis and pathogen neutralization

One of the important protective functions of TLS is to promote the production of intrathecal immunoglobulins. B cells that have completed affinity maturation within the TLS can differentiate into plasma cells and locally produce large amounts of high-affinity antibodies within the CNS [70]. These intrathecally synthesized antibodies can effectively neutralize viral particles in the CNS, preventing viral transmission and further spread of infection [68]. For example, persistent virus-specific antibody-secreting cells have been observed in the CNS following LCMV infection [70]. This local antibody production mechanism is crucial for overcoming the limitations of the blood-brain barrier and achieving effective immune protection within the CNS [67].

#### Promotes the formation of tissue-resident T cell populations

TLS also synergistically activates CD4^+^ helper T cells through the interaction of FDCs and Tfh cells [77, 78] and promotes the long-term residence and functional maintenance of CD8^+^ tissue-resident memory T cells (Trm cells) in the CNS parenchyma [73, 79, 80]. Trm cells are a special subset of memory T cells that reside in non-lymphoid tissues and provide early and strong local protection against infection and tumors [73, 80]. Peripheral immunization can generate Trm cells in the CNS, thereby providing strong local protection against infection [73]. In the CNS, Trm cells are present in both healthy and disease states, and exhibit a unique phenotype, especially in neurological diseases such as MS [81]. The long-term presence of these Trm cells in the CNS can enhance the rapid cytotoxic response to reinfection and form a lasting immune memory [73, 82], which is more helpful in preventing viral recurrence or persistent infection compared to circulating memory T cells that require re-activation in the periphery.

#### Limitations and caveats of protective TLS responses

While TLS in viral encephalitis have been associated with protective functions, several caveats warrant consideration. First, most evidence comes from animal models; direct evidence in human viral encephalitis remains limited. Second, the protective outcome likely depends on the timing of TLS formation, early formation may aid viral clearance, whereas late or persistent TLS may contribute to immunopathology. Third, the balance between protective and pathogenic effects may shift with the duration of infection: acute, self-limiting infections may resolve without sequelae, while chronic viral persistence could drive TLS maturation toward a pathogenic phenotype. Therefore, the protective role of TLS in the CNS should be interpreted within the specific context of each infectious setting.

## Pathogenic effects: TLS as a driver of neurological diseases

**Fig. 3:**
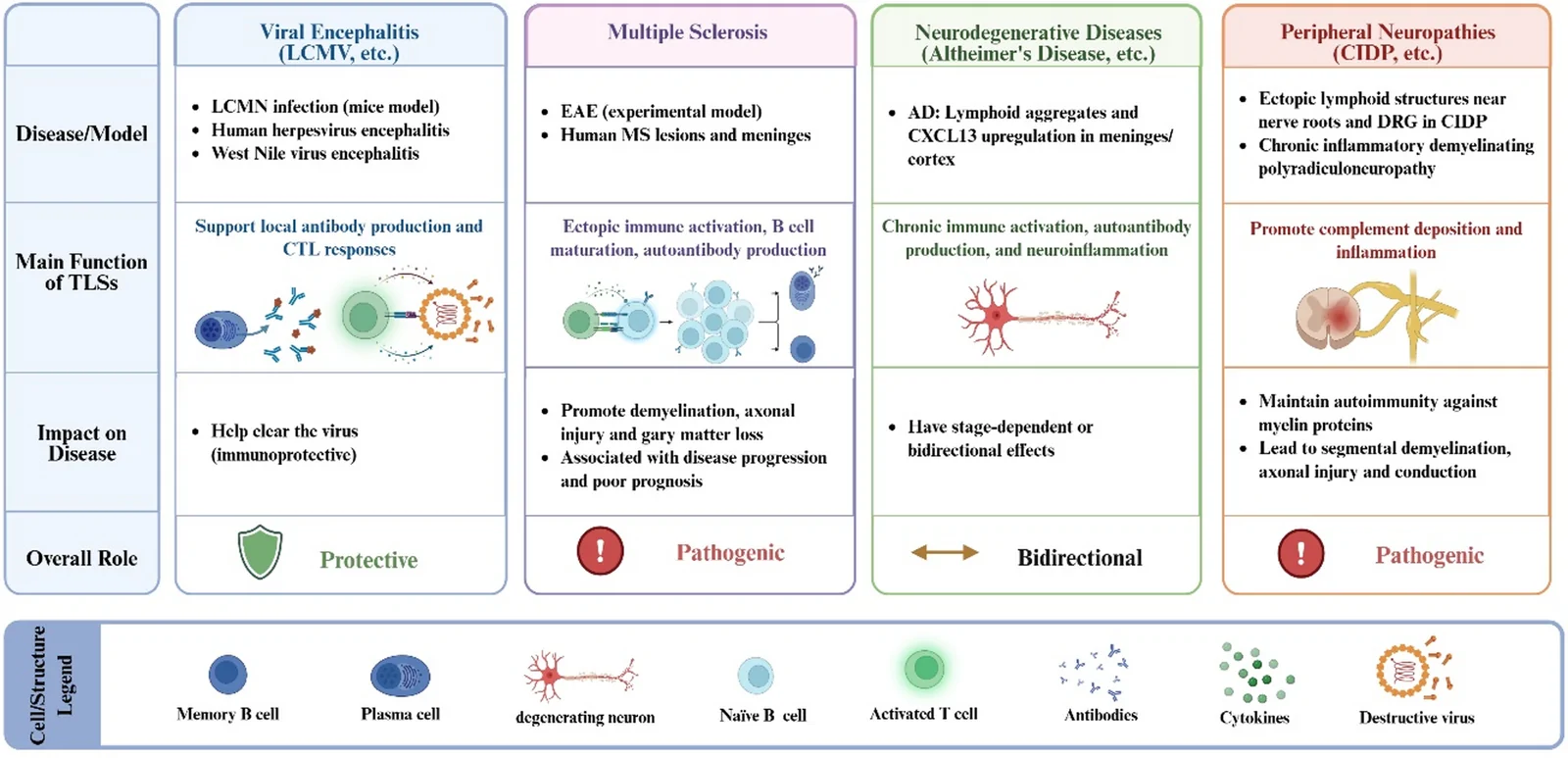
Models and evidence for TLS in neurological diseases. This figure summarizes the formation and functional roles of TLSs across different neurological disease models and clinical conditions. In viral encephalitis models, such as LCMV infection, TLSs support local antibody production and cytotoxic T lymphocyte (CTL) responses, thereby facilitating viral clearance and exerting protective immune functions. In MS, TLSs are closely associated with demyelination, axonal injury, and disease progression, predominantly contributing to pathogenic inflammation. It is usually considered as an amplifying factor of pathogenic inflammation, but its direct causal effect still needs further confirmation. In neurodegenerative disorders, including Alzheimer’s disease (AD), TLS-associated immune aggregates may exhibit bidirectional effects. In peripheral neuropathies such as chronic inflammatory demyelinating polyradiculoneuropathy (CIDP), TLSs promote local autoimmune responses and chronic inflammation, contributing to peripheral nerve injury. But its direct role in peripheral nerve injury still needs further research. Overall, TLSs may function as protective, pathogenic, or context-dependent immune structures depending on the disease setting

The pathogenic functions of TLS in neurological diseases, like their protective roles, are context-dependent and shaped by multiple interrelated factors, including the nature of the inciting antigen (self vs. non-self), anatomical location (meningeal vs. parenchymal vs. perivascular), structural maturity (lymphoid aggregates vs. fully organized TLS with germinal centers), cellular composition (effector vs. regulatory cells), disease stage (acute vs. progressive), and duration of inflammation. Moreover, TLS may exert both protective and pathogenic effects simultaneously or sequentially in the same disease. For example, in multiple sclerosis, meningeal TLS are associated with worsened outcomes, yet they might also contribute to immune surveillance or remyelination under certain conditions, possibilities that remain underexplored. The following discussion highlights the predominant pathogenic roles reported in the literature, while acknowledging the contextual nuances that shape these outcomes. In a variety of neurological diseases, the persistence of TLS is associated with uncontrolled local immune responses, tissue damage and disease progression [72, 73] (Fig. 3).

### Autoimmune diseases

#### Multiple sclerosis

In MS, the presence of bona fide TLS has been confirmed by histopathological evidence. Meningeal TLSs are a relatively common pathological feature in MS patients, especially those in the progressive or non-remission phase [30, 83, 84]. These structures meet the histological criteria for mature TLS and are located in the leptomeninges, adjacent to type Ⅲ subcortical lesions, and are associated with more severe gray matter damage and worse clinical outcomes [33]. Subsequent studies have shown that the relative degree of meningitis and the presence of TLS in MS are associated with a more severe and progressive course of disease [33, 84, 85]. The composition of immune cells in the TLS and the differences in immunoglobulin profiles in different regions of the central nervous system are the focus of current research. The pathophysiology of MS involves immune cells crossing the blood-brain barrier and working synergistically through mechanisms such as Th1/Th17 cells, B cells and the complement system [86, 87], which is accompanied by elevated levels of pro-inflammatory cytokines such as IFN-γ and TNF-α [88], leading to demyelination and axonal damage. B cells play multiple roles in the pathogenesis of MS, making them a key target for highly effective disease-modifying therapies such as anti-CD20 therapy. Meningeal TLSs can support the activation of intrathecal B cells, plasma cell differentiation, and the sustained production of high-affinity autoantibodies [89]. It is currently unclear whether the meningeal TLS in MS is a driver of disease progression, an amplifier of inflammation, a biomarker of disease activity, or a byproduct of long-term antigen stimulation. In other words, whether they are the direct cause of disease progression or a secondary result of chronic inflammation. Distinguishing these possibilities requires longitudinal studies and targeted interventions. In addition, the possibility that TLS may recruit regulatory immune cells or promote myelin regeneration under specific conditions has not been systematically ruled out.

#### Myelin oligodendrocyte glycoprotein antibody-associated disease (MOGAD) and aquaporin-4 antibody-positive neuromyelitis optica spectrum disorder (AQP4-NMOSD)

MOGAD and AQP4-NMOSD are demyelinating diseases different from MS, and they have unique pathological mechanisms and treatment strategies [90]. In MOGAD, the immune system attacks myelin oligodendrocyte glycoprotein (MOG), while AQP4-NMOSD targets aquaporin-4 (AQP4), leading to astrocyte necrosis. Although MOGAD and AQP4-NMOSD differ from MS [91, 92], the role of TLSs in their pathogenesis has gradually attracted attention. Interestingly, under steady-state conditions, the meninges actively eliminate MOG-reactive B cells through local negative selection. A study using a MOG-specific BCR knock-in mouse model demonstrated that IgM^+^ B cells in the meninges undergo apoptosis upon encountering CNS-specific antigens [93]. This finding suggests that the emergence of anti-MOG autoimmunity in MOGAD may involve the disruption of meningeal tolerance checkpoints or the formation of TLS, which could provide a microenvironment for escape selection for autoreactive B cells. In addition, leptomeningeal enhancement has been reported in patients with MOGAD and AQP4-NMOSD, but there are few studies on it in pediatric MS and pediatric AQP4-NMOSD [94]. These studies help identify the neurobiological basis of gray matter atrophy in different neuroinflammatory diseases, thereby guiding differentiated diagnostic and treatment strategies [94]. In these diseases, TLSs may exacerbate nerve damage by supporting local autoantibody production and inflammatory responses.

#### Autoimmune encephalitis

Autoimmune encephalitis is a group of diseases caused by autoantibodies attacking neuronal surface antigens. Anti-NMDAR encephalitis is one of the most widely studied types. TLSs may play a potential role in these antibody-mediated syndromes by promoting the production of local autoantibodies, leading to neuronal dysfunction and damage. Studies have reported that mature TLS structures can be seen in ovarian teratoma tissues and are considered an important source of anti-NMDAR antibody production [95]. Germinal center-like reactions within tumors can drive antibody affinity maturation, after which the antibody enters the central nervous system and triggers synaptic dysfunction. However, the evidence for TLS or TLS-like structures in CNS itself is currently limited, and their existence belongs to the speculative category (see Sect. "Graded evidence of TLS in neurological diseases" on Graded evidence). Given that TLSs are important sites of autoantibody production in other autoimmune diseases [96], suggesting that a local microenvironment may be formed to maintain antibody responses, their role in autoimmune encephalitis is also worth exploring in depth.

### Neurodegenerative diseases

#### Alzheimer’s Disease (AD)

Alzheimer’s disease is a progressive neurodegenerative disorder characterized by the accumulation of amyloid-β (Aβ) plaques and neurofibrillary tangles. In autopsy brain tissue of AD patients, some studies have reported the presence of T cells and B cells around Aβ plaques, accompanied by elevated CXCL13 expression. Clonal expansion of CD8^+^ T cells in the cerebrospinal fluid of AD patients has also been observed, suggesting adaptive immune activation [97]. However, it remains unclear whether these immune cell aggregates represent bona fide TLS, as key structural features, have not been systematically demonstrated in AD. At present, these infiltrates are best described as TLS-like lymphoid aggregates or immune cell clusters. The functional role of these aggregates remains controversial. It has been hypothesized that Aβ plaques create a local inflammatory microenvironment that could, in theory, drive the formation of TLS-like lymphoid aggregates; however, direct evidence demonstrating that Aβ directly induces TLS formation is currently lacking. Regarding the functional role of these aggregates, it has been speculated that they may contribute to neuroinflammation and neuronal loss through pro-inflammatory factors such as IL-6 and TNF. An alternative possibility is that these immune aggregates represent an epiphenomenon of chronic neurodegeneration rather than a causal driver of pathology. At present, the causal relationship between TLS-like aggregates and AD pathology has not been established, and the field remains in the hypothesis-generating stage. We therefore propose the following as a working hypothesis: Aβ-associated inflammation creates a permissive microenvironment that may support lymphoid aggregation, which could in turn modulate local immune responses; however, rigorous testing of this hypothesis awaits future studies with systematic histopathological characterization and longitudinal analysis. Given the current lack of evidence for mature TLS in AD, a biphasic or stage-dependent role of fully formed TLS remains speculative and requires further structural validation. The functional ambiguity of TLS-like aggregates in AD, potentially promoting both Aβ clearance and neuroinflammation, underscores the context-dependent nature of their roles, which may vary with disease stage and aggregate maturity.

#### Parkinson’s disease

Parkinson’s disease is another common neurodegenerative disease, characterized by the abnormal aggregation of α-synuclein. α-synuclein can serve as a potential autoantigen and can activate peripheral and central T cell responses [98]. Lymphocyte aggregation was also found in the substantia nigra and meningeal regions in the autopsy brain tissue of PD patients. However, these lymphoid aggregates lack key structural features of mature TLSs and therefore do not meet the criteria for bona fide TLS. Nevertheless, they may still contribute to the pathological process of PD through local inflammatory responses. Beyond lymphoid aggregation, infectious triggers may also contribute to neuroinflammatory and neurodegenerative processes in neurodegenerative diseases such as PD [99]. Whether these lymphoid aggregates represent an early stage of TLS formation that could eventually mature into structured TLS, or remain as simple infiltrates without functional significance, remains unknown.

We boldly propose a hypothetical model, if these lymphoid aggregates acquire higher-order organizational features such as B/T cell zoning or FDC architecture, they may participate in the pathological process of Parkinson’s disease through localized inflammatory responses. However, these aggregates are most conservatively interpreted as byproducts of neuronal damage or non-specific immune responses, rather than primary drivers of disease progression. Therefore, the role of lymphoid aggregates in Parkinson’s disease is still speculative, and the hypothesis that α-synuclein drives TLS formation still requires direct experimental verification.

#### Amyotrophic Lateral Sclerosis (ALS)

Amyotrophic lateral sclerosis (ALS) is a neurodegenerative disease that affects motor neurons. In ALS, TLS-like lymphoid aggregates have been observed in the spinal meninges, and their density is correlated with the rate of disease progression. The local inflammatory microenvironment associated with these aggregates may exacerbate motor neuron damage by releasing pro-inflammatory factors such as TNF-α and IL-6. However, it remains unclear whether these structures meet the criteria for bona fide TLS, as key features have not been systematically demonstrated in ALS. Therefore, while the presence of these TLS-like aggregates suggests that adaptive immunity may contribute to disease pathogenesis in a subset of ALS cases, evidence for fully formed TLS in ALS is currently lacking. The correlation between TLS-like aggregate density and disease progression suggests functional relevance, but whether this relationship is causal or epiphenomenal requires further investigation.

### Peripheral neuropathy

The potential effects of TLS are not limited to the central nervous system. In peripheral nervous system diseases such as chronic inflammatory demyelinating polyneuropathy (CIDP), lymphoid aggregates can be seen near nerve trunks and dorsal root ganglia [38]. However, it remains unclear whether these structures represent bona fide TLS. At present, these are best described as TLS-like lymphoid aggregates or ectopic lymphoid tissue of unknown organization. If these aggregates indeed support local autoantibody production and complement deposition, they could potentially maintain sustained attack on myelin proteins, thereby promoting segmental demyelination and axonal damage, leading to nerve conduction block and muscle atrophy. However, direct evidence linking these lymphoid aggregates to specific functional outcomes in CIDP remains to be established. The functional significance of these peripheral nerve aggregates awaits systematic structural characterization, which would help determine whether they represent a targetable pathogenic mechanism or an epiphenomenon of chronic inflammation.

### Pathogenic mechanism

TLS is ectopic lymphoid tissue that can mimic the structure and function of secondary lymphoid organs (SLOs), including the formation of germinal centers, thereby supporting the activation, proliferation, somatic hypermutation, differentiation of plasma cells, and continuous production of autoantibodies [22]. These autoantibodies can directly attack nerve cells or myelin sheaths in the central nervous system, activate the complement system, and lead to demyelination and neuronal damage. Oligoclonal bands (OCB) in the cerebrospinal fluid of MS patients are produced by clonal expansion of B cells and are a typical marker of local antibody production in the CNS. Although the targets of these antibodies are not clearly defined, they can recognize myelin and non-myelin components, and some of them also have cross-reactivity with EBV [32]. In addition to autoantibodies, TLS can also produce a large number of pro-inflammatory cytokines locally, such as IL-6, TNF, IFN-γ, etc. These cytokines can exacerbate the local inflammatory response, recruit more immune cells into the lesion area, form a vicious cycle, and maintain the inflammatory circuit [96, 100]. In MS, B cells from blood, CSF, and CNS lesions do indeed produce pro-inflammatory factors such as Granulocyte-Macrophage Colony-Stimulating Factor (GM-CSF), IL-6, and TNF, which promote the activation and polarization of myeloid T cells; in particular, meningeal B cells induce T cells to produce GM-CSF through MHC class II-dependent interactions, thereby triggering neutrophil recruitment and endothelial activation, amplifying inflammatory recurrence [32].

Epitope expansion refers to the process by which the target of an immune response spreads from the initial self-antigen to a new self-antigen [22]. The germinal-like reaction within TLS can provide an ideal microenvironment for this epitope extension. Under conditions of chronic inflammation, persistent exposure to self-antigens can be captured by local antigen-presenting cells (APCs) and presented to T cells and B cells, thereby activating an immune response against these new epitopes. This phenomenon is particularly important in autoimmune diseases because it means that the scope of the disease’s immune attack expands over time, leading to more extensive tissue damage and disease progression.

**Fig. 4:**
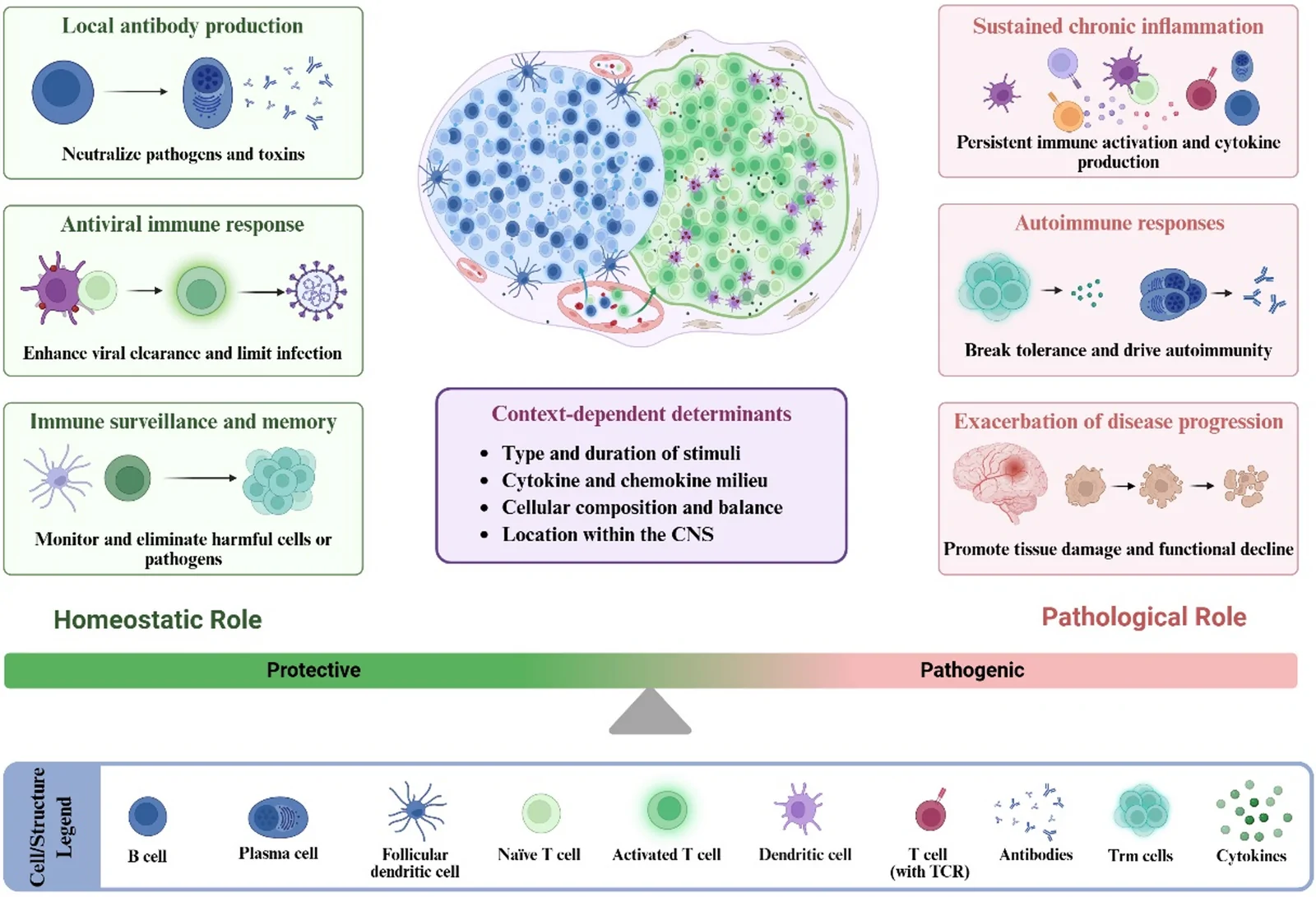
Dual role of TLS in neuroinflammation. TLSs exert both protective and pathogenic functions during neuroinflammatory processes. The left side illustrates their protective and homeostatic roles, including local antibody production, antiviral immune responses, immune surveillance, and immune memory formation, all of which contribute to pathogen clearance and maintenance of tissue homeostasis. The right side depicts their pathogenic roles, including sustained chronic inflammation, amplification of autoimmune responses, and exacerbation of disease progression, ultimately leading to tissue damage and neurological dysfunction. The pathogenic effect on the right side is mainly based on the correlation evidence between TLS and disease severity, and its direct causal role in disease progression still needs further verification. TLSs are positioned centrally as a functional hub, highlighting their context-dependent nature, which is influenced by stimulus type and duration, cellular composition, cytokine and chemokine milieu, and anatomical localization within the CNS

The presence and activity of TLS can alter the homeostasis of the local microenvironment. For example, chemokine networks such as CXCL13/CXCR5 and CCL19/CCR7 may lead to the disruption of the blood-brain barrier or neural barrier, causing continuous recruitment of lymphocytes and affecting the homeostasis of glial neurons, thereby promoting neuroinflammation and neuronal damage [17, 40]. Chronic inflammation and changes in the chemokine network may impair the integrity of the blood-brain barrier or blood-nerve barrier [101–103]. Impaired barrier function makes it easier for circulating immune cells, inflammatory mediators, and autoantibodies to enter nerve tissue, thereby further aggravating local inflammation and nerve damage. In addition, the pro-inflammatory cytokines and autoantibodies produced within TLS can influence the activity and function of glial cells [22]. Activated microglia and astrocytes play a dual role in neurological diseases: they can both clear harmful substances and release pro-inflammatory mediators, exacerbating neuroinflammation and neuronal damage. By maintaining a local inflammatory environment, TLS may promote the transformation of glial cells to a continuously activated pro-inflammatory phenotype, thereby disrupting the normal neuronal microenvironment and leading to synaptic dysfunction and neurodegeneration (Fig. 4).

## Diagnostic and therapeutic value of targeting TLS

TLS is considered to be ectopic lymphoid tissue that forms in the chronic inflammatory microenvironment and plays an important role in a variety of central nervous system diseases. Especially in autoimmune diseases such as multiple sclerosis, meningeal TLS is closely associated with cortical demyelination, persistent inflammation, and disease progression. With a deeper understanding of the occurrence and functional mechanisms of TLS, diagnostic biomarkers and targeted intervention strategies for TLS have become an important direction for precision neuroimmunotherapy.

### Biomarkers

#### Imaging: current limitations and future prospects of PET and high-resolution MRI

It should be noted that there is currently no research that directly uses PET or high-resolution MRI for specific imaging of TLS in neurological diseases. Therefore, the following discussion is purely prospective and aims to point out potential future research directions based on indirect evidence, rather than describing established diagnostic capabilities. We can infer its potential from relevant research. For example, MRI is widely used to identify lesions in the diagnosis of neurological diseases. High-resolution MRI can be used to observe inflammatory activity in the central nervous system in diseases such as multiple sclerosis [104]. In the future, it may be possible to develop methods to identify the presence of TLS by recognizing meningeal/pial enhancement and abnormal signals in subcortical white matter. However, such methods are still speculative and need to be validated against the gold standard of histopathology before they can be established. PET imaging technology, especially with the use of specific radioactive tracers, has been used to visualize and measure neuroinflammation in neurodegenerative diseases such as Alzheimer’s disease. For example, in the neuropsychiatric manifestations of systemic lupus erythematosus, the brain TLS has been described, suggesting its potential role in disease progression [105]. In the future, a CXCL13-PET probe may be developed for TLS-specific imaging. However, such probes have not yet been developed and have not been validated in the human body. These advanced imaging technologies hold promise for non-invasive detection and localization of TLS in vivo. However, their current applications are still hypothetical. The imaging detection of TLS from conception to clinical or research applications still requires a lot of technological development and validation research related to histopathology.

#### Cerebrospinal fluid analysis: CXCL13 and antibody profile

Cerebrospinal fluid analysis plays an irreplaceable role in the diagnosis and monitoring of neurological diseases. CXCL13 is a chemokine that specifically binds to CXCR5 [106, 107]. In neurological disorders, particularly MS, elevated levels of CXCL13 in cerebrospinal fluid are considered a reliable biomarker for TLS activity [107, 108]. CXCL13 can promote the migration of B cells and Tfh cells to B cell follicles, thereby promoting the interaction between B cells and T cells and antibody formation [107]. Significantly elevated CXCL13 concentrations in cerebrospinal fluid, combined with the detection of IgG index, oligoclonal bands (OCB), and anti-AQP4/NMDAR antibody profiles, can improve the accuracy of patient stratification, such as predicting relapse risk and treatment response. This is especially important for diseases such as MS that require early and efficient treatment, as CXCL13 can serve as a molecular biomarker for optimizing immunotherapy [109].

#### Patient stratification potential

Combining imaging evidence with cerebrospinal fluid biomarkers can more accurately stratify patients. For example, in the field of oncology, the presence and maturity of TLS have been shown to be closely related to patient prognosis and response to immune checkpoint inhibitor (ICI) therapy [110–112]. In non-small cell lung cancer (NSCLC), the presence of CXCL13-positive T cells in the tumor microenvironment is a good predictor of ICI response [113]. Mature TLS, regardless of PD-L1 expression levels, can independently predict the efficacy of immune checkpoint inhibitors [110]. This suggests that combining multiple biomarkers can identify patient groups most likely to benefit from specific treatments, thus enabling precision medicine.

### Treatment strategies

Treatment strategies for TLS can be broadly categorized into two types: inhibiting its formation or function to treat autoimmune diseases, and modulating it without disrupting protective immune surveillance, particularly in neurodegenerative diseases.

#### Suppress TLS formation/function

The CXCL13/CXCR5 signaling axis plays a key role in immune cell recruitment and activation as well as adaptive immune regulation [106]. The binding of CXCL13 to CXCR5 promotes the migration of B cells and follicular helper T cells to B cell follicles, leading to the formation of TLS [107]. In autoimmune diseases, such as lupus nephritis, TLS is considered a local persistent factor leading to organ-specific immune damage [96]. Drugs that block the CXCL13/CXCR5 axis can theoretically reduce the formation of TLS or disrupt its function by inhibiting lymphocyte infiltration. For example, in a classic model of neuropsychiatric lupus (NPSLE), neutralization of CXCL13 reduced memory impairment and depressive-like behaviors [105].

B cells are an important component of the immune response to TLS and play a key role in the immune response to a variety of autoimmune diseases and certain tumors [114]. Anti-CD20 monoclonal antibodies such as rituximab, ocrelizumab and ofatumumab can lead to B cell depletion [104]. In autoimmune diseases, these therapies can effectively inhibit disease progression by reducing the production of germinal center-like structures and pathogenic antibodies within the TLS. In the treatment of multiple sclerosis, anti-CD20 is a highly effective disease-modifying therapy [115].

#### Regulation rather than elimination

While it is desirable to suppress TLS in autoimmune diseases, TLS may also play a beneficial role in certain situations, such as in antitumor immunity. TLS is associated with good tumor prognosis and treatment response to immune checkpoint inhibitors by promoting immune cell infiltration and activating antitumor immune function [111, 116]. In patients with hepatocellular carcinoma who received neoadjuvant immune checkpoint blockade (ICB) therapy, intratumoral TLS was associated with improved prognosis, and CD20^+^ CXCL13^+^ lymphocyte clusters (LAs) were identified as potential predictors of immunotherapy response [117]. Therefore, the challenge is how to preserve or even enhance protective immune surveillance while destroying pathogenic TLS, especially in neurodegenerative diseases such as Alzheimer’s disease and glioma, where TLS may promote antigen presentation and T cell activation [118]. As a result, future treatment strategies may need to be more refined.

#### Security considerations for therapeutic interventions of TLSs or TLS-like structures

Given the dual protective and pathogenic role of TLS in neurological diseases, as mentioned in Sects. "Protective role: the role of TLS in central nervous system immune surveillance and infection control" and "Pathogenic effects: TLS as a driver of neurological diseases" above, therapeutic interventions targeting TLSs and TLS-like structures may pose a range of safety risks that must be carefully evaluated. Firstly, inhibiting TLS or TLS like structures may weaken protective antiviral immunity. In infectious diseases such as viral encephalitis, although there is no direct evidence of TLS formation in human patients at present, animal model research shows that lymphocyte aggregates or TLS-like structures may contribute to pathogen clearance and local immune response. By blocking the CXCL13/CXCR5 axis or clearing B cells, systemic intervention in these structures may impair the central nervous system’s antiviral immune defense, and even lead to virus reactivation or chronicity. Secondly, clearing TLS may disrupt anti-tumor immune surveillance. TLS is associated with anti-tumor immune response and better prognosis in brain tumors such as gliomas [28, 119, 120]. Non-selective inhibition of TLS formation or function may weaken the immune system’s ability to recognize and clear tumor cells, thereby offsetting the therapeutic benefits of immune checkpoint inhibitors. Thirdly, interventions targeting TLS may have widespread systemic effects. The CXCL13/CXCR5 signaling axis and B cell function not only participate in the formation of TLS, but also in the normal immune response of secondary lymphoid organs [121]. Systemic blockade of these pathways may lead to systemic immune suppression, increase the risk of infection, or interfere with normal immune surveillance functions. Future treatment strategies should not pursue complete clearance of TLS, but instead focus on developing more selective intervention methods, such as local targeted drug delivery, regulating the maturation status of TLS rather than clearance, or staged interventions based on disease stage and functional orientation of TLS. The security window of these strategies still needs to be systematically evaluated in preclinical models and early clinical trials.

### Future directions for clinical trials of patient selection and combination therapies based on TLS biomarkers

Future clinical trials will place greater emphasis on TLS-based biomarker-based patient selection and combination therapies to achieve more personalized and effective treatment. Combining multiple biomarkers, such as cerebrospinal fluid CXCL13 levels, MRI TLS scores, and single-cell transcriptome characteristics, will help to more accurately screen and enrich patients suitable for specific treatment regimens [122]. Further exploration of combination therapies and fine-tuning of TLS function, rather than single-method elimination, is possible, with the aim of maintaining or enhancing beneficial immune surveillance function while suppressing pathological immune responses.

**Fig. 5:**
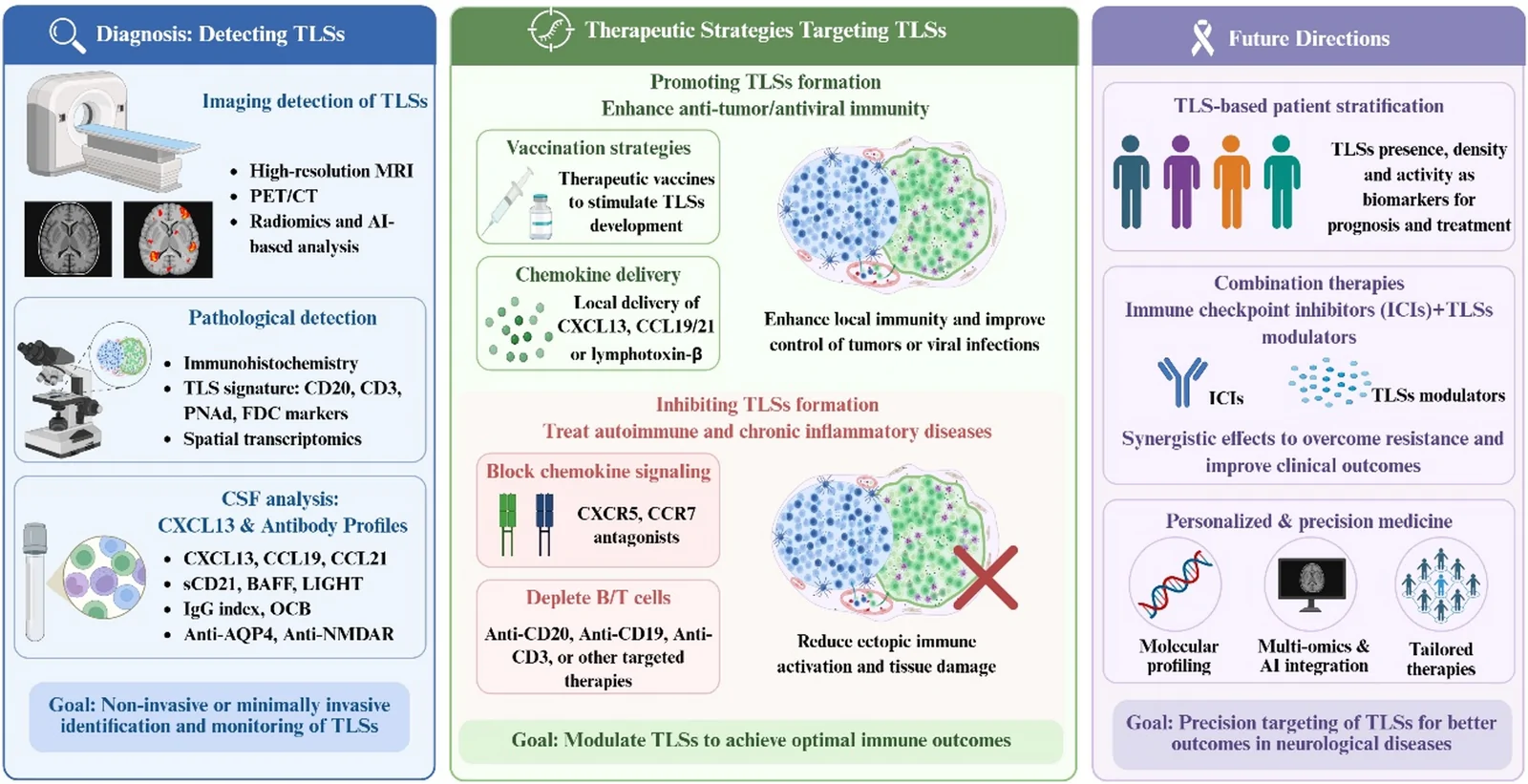
Diagnostic and therapeutic strategies and future directions for targeting TLS. TLSs can be detected through pathological analysis, and non-invasive methods such as imaging and CSF analysis are being actively explored as potential detection methods. Therapeutically, TLSs may be promoted to enhance anti-tumor or antiviral immunity via vaccines and chemokine delivery, or inhibited to treat autoimmune and chronic inflammatory diseases via blocking chemokine signals or depleting B/T cells. Future directions include TLS-based patient stratification and combination therapies integrating immune checkpoint inhibitors with TLS modulators to achieve precision immunotherapy

In summary, TLS plays a complex and diverse role in a variety of diseases. A deeper understanding of its formation, function, and regulatory mechanisms, combined with advanced biomarkers and imaging technologies, holds promise for developing more precise and effective diagnostic and treatment strategies (Fig. 5).

## Controversy and unknowns

Although TLS has been repeatedly reported in various neurological diseases, its hierarchical position and functional attributes in the development of these diseases are still not fully understood. The key questions nowadays are no longer limited to whether TLS exists, a question we have addressed in the introduction section by grading evidence across diseases, but rather revolve around its causality, disease heterogeneity, and systemic regulatory mechanisms.

### Classifying TLS and TLS-like structures in neurological diseases

When delving into the association between TLS and disease pathology, distinguish the different roles that TLS or TLS-like structures may play. Driver: TLS directly initiates or maintains pathological processes. Usually, causal evidence from longitudinal studies or intervention studies is required. Amplifier: TLS exacerbates existing inflammatory responses, but is not necessary for disease initiation. TLS is most likely to play such a role in many chronic neurological diseases. Biomarker: TLS is associated with disease severity or progression, but may not necessarily have a causal contribution. This role has reference value for prognosis assessment, but it does not necessarily mean it can be used as a therapeutic target. Epiphenomenon: TLS is formed as a secondary result of chronic inflammation or tissue damage, but has no significant independent functional impact on disease outcomes. We can use this framework to evaluate the role of TLS in various neurological disorders.

### Causality and correlation

In multiple sclerosis, meningeal TLS is closely associated with cortical demyelination and disease progression [123], suggesting that it may serve as a maintenance structure for compartmentalized inflammation. However, it is currently uncertain what role TLS plays in MS: is it a disease driver, amplifier, or simply a biomarker or epiphenomenon of chronic inflammation?

Correlation does not equate to causation. TLS is more common in the disease progression stage and may also be a secondary phenomenon of chronic antigen stimulation and persistent inflammation. A more reasonable model now suggests that there is a positive feedback loop between TLS and chronic inflammation: persistent inflammation induces the regeneration of lymphoid organs, while mature TLS, in turn, strengthens local humoral and cellular immune responses. In this model, TLS is more likely to play the role of an amplifier rather than an initial driver. Future research should focus on longitudinal imaging biomarkers and interventions targeting lymphoid organ regeneration pathways to clarify its causal role.

### TLS heterogeneity and maturation status

TLS and TLS-like structure in different diseases may have structural and functional differences, these differences may reflect the different maturity states of TLS. In multiple sclerosis, TLS typically exhibits germinal center-like, features FDCs, and HEVs, and is associated with intrathecal IgG synthesis^80^; representing the mature/highly organized state of TLS differentiation and making it more likely to act as an amplifier for inflammation; while in Alzheimer’s disease, more loosely structured lymphoid aggregates are observed^89^, and it remains unclear whether they possess mature follicular function. These TLS-like structures are currently more likely to be byproducts or biomarkers of disease processes rather than driving factors. The lymphoid aggregates observed in Parkinson’s disease, ALS, and CIDP may be in the earlier stages of this continuum. However, it is currently unclear whether different maturity states represent significant differences in TLS functionality or simply reflect differences in sampling time points. Therefore, TLS may not be a single biological entity, but rather a continuous spectrum of immune structures at different stages of maturity or shaped by disease-specific antigenic environments. Different antigenic environments may determine their functional orientation. Clarifying this heterogeneity will determine whether TLS has therapeutic value across diseases.

### Systemic regulation and age-related factors

Whether the gut-microbe axis provides remote signals for the formation of central TLS is currently lacking direct mechanistic evidence. Furthermore, it remains unclear whether immunosenescence alters the TLS formation threshold, structural maturity, or inflammatory intensity in age-related diseases such as Alzheimer’s and Parkinson’s. Meanwhile, it remains unknown whether TLS in older individuals has a higher pathogenic potential. Future research needs to combine aging immunology with TLS biology to elucidate whether and how age factors regulate the initiation, maturation, and functional orientation of TLS in the CNS.

## Conclusion

This review systematically explores the complex role of tertiary lymphoid structure (TLS) in neuroimmune interactions, particularly their duality in the context of neurological health and disease. We emphasize that TLS is not simply a pathological product, but a key interface connecting adaptive immunity and chronic neuroinflammation.

On the one hand, TLS has shown important protective effects in immune surveillance and infection control of the central nervous system. In models such as viral encephalitis, TLS can promote the local clonal expansion and affinity maturation of antigen-specific B cells by providing a germinal center-like microenvironment, thereby achieving the continuous synthesis of intrathecal high-affinity antibodies and effectively neutralizing pathogens. At the same time, TLS also promotes the formation of tissue-resident memory T cell populations, providing durable immune protection for the CNS.

On the other hand, TLS also plays a disease-driving role in a variety of neurological disorders. In multiple sclerosis, autoimmune encephalitis, and neurodegenerative diseases such as Alzheimer’s disease and Parkinson’s disease, the abnormal formation and persistence of TLS are closely related to uncontrolled local immune responses, autoantibody production, release of pro-inflammatory cytokines, tissue damage, and disease progression.

Given the dual role of TLS in neurological diseases, future treatment strategies need to move beyond traditional broad immunosuppression and towards precise regulation of the TLS-specific pathway. This means we need to develop interventions that can distinguish between protective TLS and pathogenic TLS, effectively suppressing pathological TLS while preserving or even enhancing beneficial immune surveillance functions.

To accurately elucidate the regulatory mechanisms of TLS, it is urgent to conduct in-depth longitudinal human studies, combining advanced imaging technologies (such as PET and high-resolution MRI) and cerebrospinal fluid biomarkers (such as CXCL13 and antibody profiles) to monitor the dynamic changes and functional status of TLS. Simultaneously, we will develop more precise in vitro and in vivo models to elucidate the molecular mechanisms of TLS formation, maintenance, and regulation, and evaluate the efficacy and safety of innovative therapies targeting TLS. Through these efforts, we hope to provide more precise and effective strategies for the diagnosis and treatment of neurological diseases.

## Data Availability

No datasets were generated or analysed during the current study.

## Abbreviations

CNS: Central nervous system
TLSs: Tertiary lymphoid structures
BBB: Blood-brain barrier
CAMs: Central nervous system-associated macrophages
BAMs: Boundary-associated macrophages
MLVs: Meningeal lymphatic vessels
SLOs: Secondary lymphoid organs
FDCs: Follicular dendritic cells
GCs: Germinal centers
HEVs: High endothelial venules
Tfh: Helper T cells
PNAd: Peripheral lymph node addressin
FRCs: Fibroblast reticulocytes
DCs: Dendritic cells
LT: Lymphotoxin
LTi-like cells: Lymphoid tissue inducer-like cells
LTβR: Lymphotoxin β receptor
MS: Multiple sclerosis
PNS: Peripheral nervous system
DRG: Dorsal root ganglion
CIDP: Chronic inflammatory demyelinating polyneuropathy
CSF: Cerebrospinal fluid
GM-CSF: Granulocyte-Macrophage Colony-Stimulating Factor
LTi cells: Lymphoid tissue-induced cells
cCD1: Type 1 dendritic cells
Aβ: β-amyloid
LTo: Lymphoid stromal cells
LCMV: Lymphocytic choriomeningitis virus
Tfr cells: Follicular regulatory T cells
Trm cells: Tissue-resident memory T cells
MOGAD: Myelin oligodendrocyte glycoprotein antibody-associated disease
AQP4-NMOSD: Aquaporin-4 antibody-positive neuromyelitis optica spectrum disorde
MOG: Myelin oligodendrocyte glycoprotein
AQP4: Aquaporin-4
AD: Alzheimer’s disease
PD: Parkinson’s disease
ALS: Amyotrophic lateral sclerosis
OCB: Loigoclonal bands
APCs: Antigen-presenting cells
ICI: Immune checkpoint inhibitor
NSCLC: Non-small cell lung cancer
ICB: Immune checkpoint blockade
